# The Emerging Role of UCHL1 in Neurological and Musculoskeletal Diseases

**DOI:** 10.1111/imcb.70151

**Published:** 2026-08-04

**Authors:** Ru Feng, Xiu Zhong, Jianjun Li, Adrian A. Achuthan

**Affiliations:** ^1^ Department of Medicine Royal Melbourne Hospital, The University of Melbourne Parkville Victoria Australia; ^2^ School of Rehabilitation Capital Medical University Beijing China; ^3^ Department of Spinal and Neural Functional Reconstruction China Rehabilitation Research Center Beijing China; ^4^ Chinese Institute of Rehabilitation Science Beijing China

**Keywords:** cancer, deubiquitinating enzyme, inflammation, musculoskeletal conditions, neurological disorders, signaling pathways, UCHL1

## Abstract

Ubiquitin C‐terminal hydrolase L1 (UCHL1) is a highly conserved deubiquitinating enzyme that has transitioned from being viewed as a “brain‐specific” protein to a global regulator of cellular proteostasis and signal transduction. As a key component of the ubiquitin‐proteasome system (UPS), UCHL1 maintains the intracellular free ubiquitin pool through its C‐terminal hydrolase activity, while also exhibiting atypical ligase‐like functions and acting as a molecular scaffold for signaling complexes. Beyond its classical role in neurons, increasing evidence suggests that UCHL1 participates in diverse pathological conditions including neurodegeneration, cancer, cardiovascular and metabolic diseases, as well as musculoskeletal disorders. Through regulation of protein turnover, oxidative stress, inflammatory signaling, and cell survival, UCHL1 emerges as a context‐dependent regulator with dual protective and pathogenic roles. This review explores the sophisticated multi‐level regulation of UCHL1, ranging from transcriptional control to epigenetic silencing and posttranslational modifications, summarizes the recent research progress of UCHL1 in various systems, and elaborates on its mechanism of action in various conditions, including neurological and musculoskeletal disorders.

## Graphical abstract

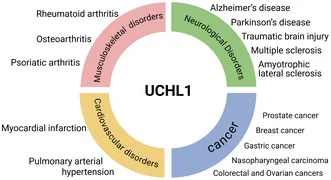

## 1 Introduction

The ubiquitin–proteasome system (UPS) serves as a primary mechanism for maintaining cellular proteostasis and orchestrating intracellular signaling networks. Ubiquitination dictates protein fate through the architectural assembly of mono‐ or polyubiquitin chains; typically, K48‐linked chains target substrates for proteasomal degradation, whereas K63‐linked chains predominantly mediate nonproteolytic processes, including endocytosis, DNA repair, and signal transduction [1, 2]. This dynamic modification is counterbalanced by deubiquitinating enzymes (DUBs), which remove ubiquitin moieties from substrates, edit chain architecture, replenish the free ubiquitin pool, and modulate the stability or activity of downstream signaling targets [3, 4]. While the human genome encodes nearly 100 distinct DUBs categorized into structurally diverse families, their collective biological significance resides in their capacity to finely tune protein turnover, immune signaling, cellular stress responses, and disease‐associated molecular pathways [5, 6].

Within this superfamily, ubiquitin C‐terminal hydrolase L1 (UCHL1) is distinctive due to its capacity to link baseline ubiquitin homeostasis with tissue‐specific signaling cascades. UCHL1 belongs to the structurally defined ubiquitin C‐terminal hydrolase family alongside UCHL3, UCHL5, and BRCA1‐associated protein 1 (BAP1) [7]. Initially characterized as a neuron‐enriched protein, UCHL1 is abundantly expressed in neurons and neuroendocrine cells, where it maintains the intracellular free ubiquitin pool to sustain axonal integrity and synaptic function [8]. While this high neural abundance has led to its widespread clinical use as a biomarker of central nervous system injury, accumulating evidence indicates that UCHL1 expression and function are not restricted to the nervous system. Rather, UCHL1 actively participates in oncogenesis, cardiovascular remodeling, metabolic stress, immune activation, and musculoskeletal disorders, establishing it as a multifaceted regulator of proteostasis and pathological signaling across diverse tissue contexts [9].

The regulatory functions of UCHL1 are based on several interconnected mechanisms. First, UCHL1 preserves the intracellular free ubiquitin pool and maintains proteostasis via its canonical C‐terminal hydrolase activity [10, 11]. Second, it stabilizes disease‐associated substrates and downstream signaling mediators through targeted deubiquitination, thereby modulating pathways governing protein aggregation, oxidative stress, inflammation, hypoxia, cellular metabolism, and survival [12]. Third, its expression and functional activities are tightly controlled by upstream transcriptional networks, epigenetic modifications, noncoding RNAs, posttranslational modifications, and subcellular localization. Finally, accumulating evidence suggests that UCHL1 exerts noncanonical functions distinct from classical substrate deubiquitination. Collectively, these multifaceted attributes explain why UCHL1 exhibits highly divergent, context‐dependent effects across pathologies, functioning as a protective factor, a pathogenic driver, or a passive biomarker depending on the specific cell type and microenvironmental stimulus.

In this review, we first summarize the biochemical and regulatory foundations of UCHL1, including its integration within the UPS, its roles in substrate deubiquitination and ubiquitin homeostasis, and its upstream regulation via posttranslational modifications and noncanonical activities. We then examine the expanding body of evidence linking UCHL1 to neurological disorders, oncogenesis, cardiovascular conditions, and musculoskeletal diseases. By organizing this review sequentially from fundamental molecular mechanisms to clinical pathology, we aim to resolve the context‐dependent functions of UCHL1 and outline how these distinct modalities can better inform future therapeutic strategies and biomarker development.

## 2 Regulatory Functions of UCHL1

**FIGURE 1:**
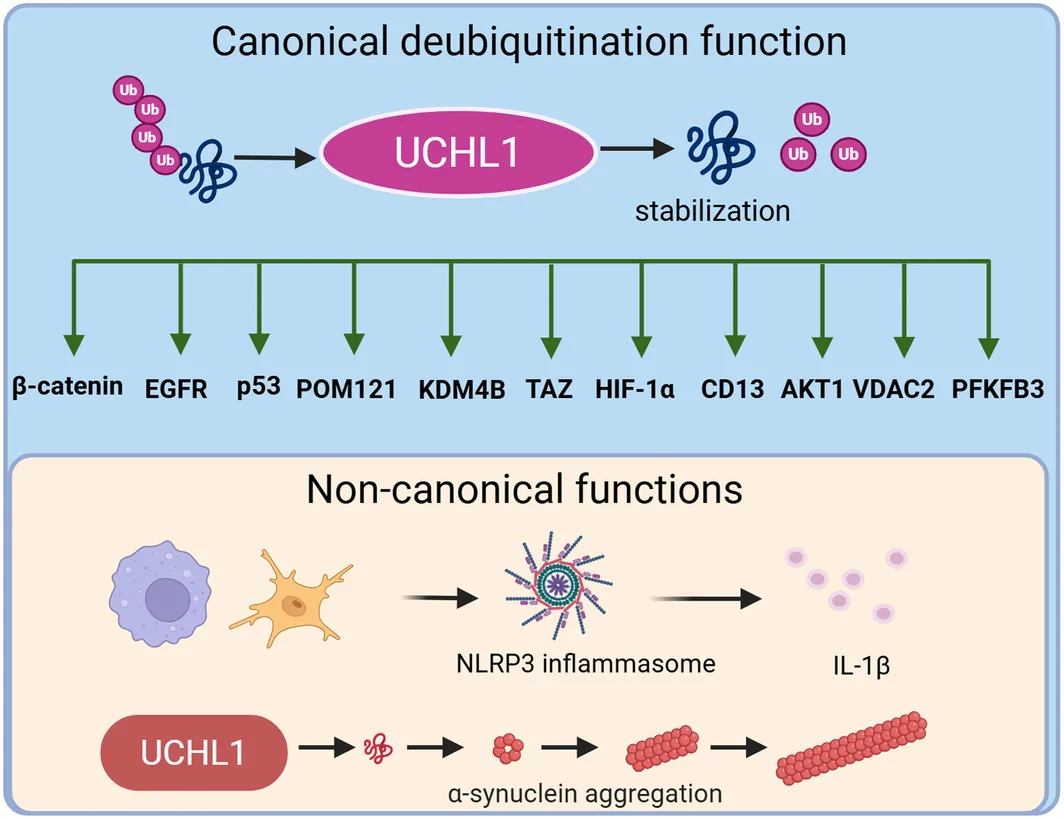
Regulatory functions of UCHL1. Canonical and noncanonical regulatory functions of UCHL1 activate several signaling pathways and biological processes in various diseases.

UCHL1 modulates downstream signaling networks primarily through substrate deubiquitination and stabilization, thereby altering the cellular abundance and activity of target proteins (Figure 1). Beyond its canonical deubiquitination function, UCHL1 also influences cell signaling by maintaining ubiquitin homeostasis, altering protein localization, executing potential ligase‐like activity, and directly associating with macromolecular signaling complexes.

### 2.1 Canonical Deubiquitination Function

The canonical deubiquitinating activity of UCHL1 regulates diverse cellular processes by directly interacting with and stabilizing target proteins. For instance, the endogenous association of UCHL1 with β‐catenin prevents its degradation, thereby enhancing β‐catenin/TCF‐dependent transcription [4]. In models of cardiac hypertrophy and cardiovascular remodeling, UCHL1 deubiquitinates and stabilizes the epidermal growth factor receptor (EGFR), and this phenotype can be reversed via UCHL1 knockdown or pharmacological inhibition with LDN‐57444 [13]. In oncogenic contexts, UCHL1 interacts with and stabilizes the nuclear pore component POM121, preserving neuroendocrine identity and facilitating the POM121‐dependent nuclear translocation of E2F1 and c‐MYC to accelerate cancer progression [14]. In clear cell renal cell carcinoma, wild‐type UCHL1, but not its catalytically inactive C90A mutant, deubiquitinates and stabilizes lysine demethylase 4B (KDM4B), which upregulates VEGF signaling and confers therapeutic resistance to bevacizumab [15]. UCHL1 also plays a complex role in tumor suppression pathways by deubiquitinating p53 and p14(ARF) resulting in p53 stabilization and the activation of downstream p53 signaling pathway in nasopharyngeal carcinoma pathogenesis [16]. Conversely, in other malignancies, UCHL1 enhances metastatic potential by preventing the von Hippel–Lindau‐driven ubiquitination and degradation of hypoxia‐inducible factor‐1α (HIF‐α) thereby stabilizing this crucial regulatory subunit [17].

This regulatory control over HIF‐1α is also vital in nonmalignant tissues. Within the cartilage microenvironment, UCHL1‐mediated stabilization of HIF‐1α activates the HIF‐1α‐SOX9 axis and downregulates MMP13, which enhances the chondrogenic differentiation and matrix synthesis of mesenchymal stem cells [18]. Concurrently, UCHL1 mitigates chondrocyte apoptosis by promoting HIF‐1α‐dependent mitophagy to maintain mitochondrial homeostasis, underscoring its protective role in osteoarthritis (OA) [19]. Recent investigations have also uncovered a sophisticated negative feedback loop composed of the RANKL‐MAPK‐UCHL1‐sCD13 axis; here, UCHL1 stabilizes CD13 and promotes the shedding of soluble CD13, which suppresses MAPK signaling and osteoclastogenesis to delay OA progression [20]. UCHL1 also acts as a critical checkpoint in bone homeostasis and cellular differentiation. Although UCHL1 is upregulated during RANKL‐induced osteoclastogenesis, it deubiquitinates and stabilizes TAZ, which suppresses NFATc1 and its downstream effector genes to ultimately inhibit osteoclast differentiation [21].

In reproductive biology, UCHL1 deubiquitinates and stabilizes voltage‐dependent anion channel 2 (VDAC2), thereby enhancing the synthesis of pregnenolone—the precursor of estradiol [22]. Finally, UCHL1 maintains the stability of AKT1, as its inhibition or ablation accelerates the K48‐linked ubiquitination and proteasomal degradation of AKT1, thereby suppressing endothelial proliferation and pulmonary vascular remodeling [23]. Similarly, UCHL1 stabilizes 6‐phosphofructo‐2‐kinase/fructose‐2,6‐bisphosphatase 3 (PFKFB3) via deubiquitination, which stimulates glycolysis in astrocytes, sustains metabolic reprogramming, and inhibits neuronal ferroptosis to exert robust neuroprotective effects [24].

### 2.2 Noncanonical Regulatory Functions

Not all reported functions of UCHL1 can be categorized as classical, substrate‐specific deubiquitination. Crucially, a given UCHL1 function should be classified as truly DUB‐independent only when it is preserved following complete catalytic inactivation validated by catalytic‐dead mutants, activity‐deficient rescue experiments, or physical binding assays that demonstrate a scaffold‐like activity independent of ubiquitin hydrolysis. While numerous published studies demonstrate that UCHL1 interacts with macromolecular signaling complexes, alters protein subcellular localization, or modulates downstream pathway activation, the absolute dependence of these phenotypes on its enzymatic activity has not always been directly evaluated. Consequently, until strict DUB independence is experimentally substantiated, these phenomena are more accurately described as noncanonical activities that operate beyond classical deubiquitination (Figure 1).

Notably, UCHL1 interacts with the NACHT domain of the NLRP3 inflammasome, regulating its macromolecular assembly and subsequent IL‐1β production in human macrophages and microglia, and this noncanonical interaction provides a unified molecular basis connecting joint synovitis and neuroinflammation [12]. Beyond scaffolding functions, it has been proposed that UCHL1 can exhibit a distinct ligase‐like activity upon homodimerization, which promotes the accumulation of α‐synuclein through a mechanism distinct from its baseline hydrolytic activity [25]. However, this putative ligase function remains mechanistically debated, and its operational relationship to the catalytic cysteine residue and classical hydrolase activity requires careful interpretation. Furthermore, posttranslational modifications heavily dictate these noncanonical roles; for example, C‐terminal farnesylation actively promotes the membrane association of UCHL1 and alters its intracellular interaction network, thereby accelerating α‐synuclein accumulation and subsequent neurotoxicity [26].

## 3 Molecular Regulation of UCHL1

### 3.1 Transcriptional Regulation

**FIGURE 2:**
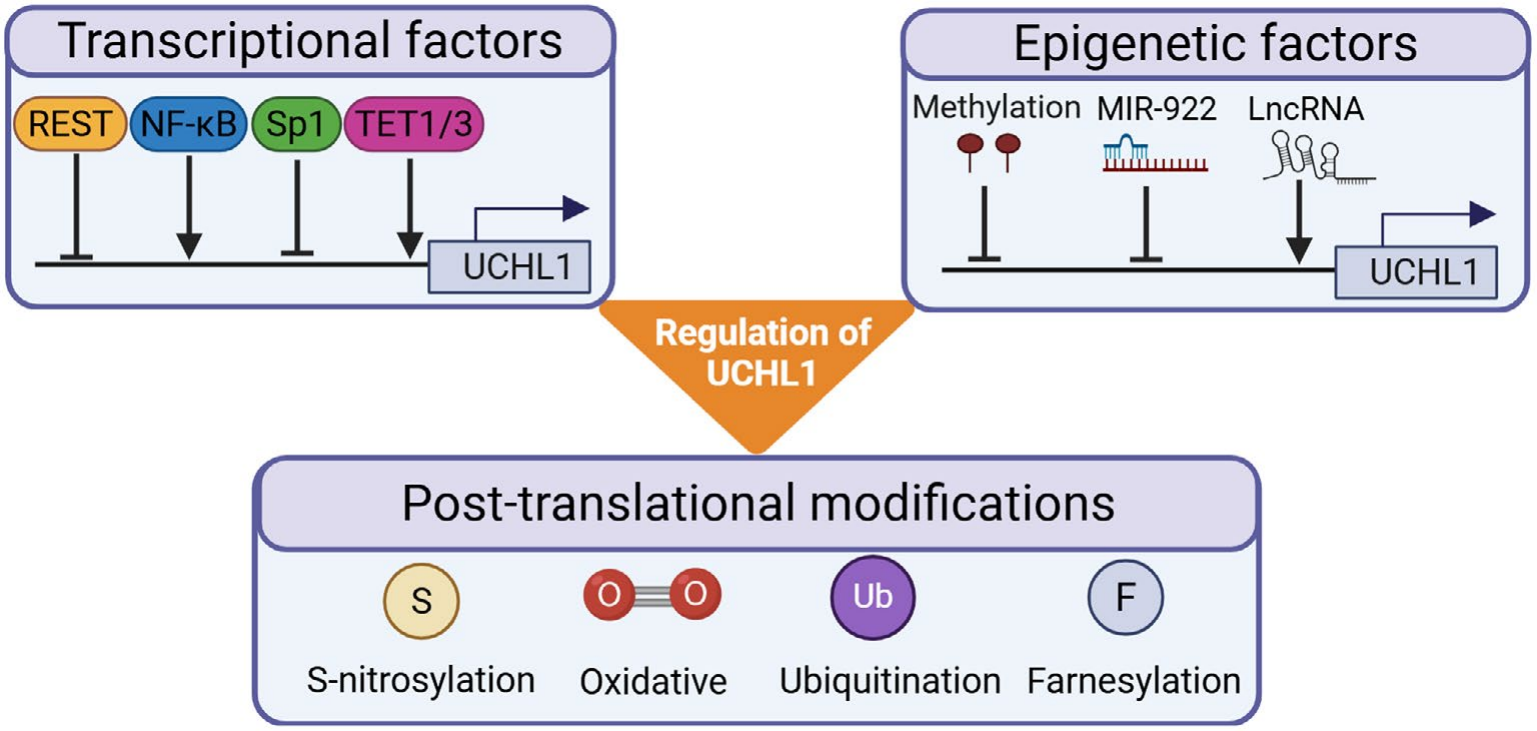
Molecular regulation of UCHL1. Expression and function of UCHL1 are regulated by transcriptional and epigenetic factors as well as posttranslational modifications.

Several upstream transcription factors and epigenetic modifiers tightly control UCHL1 expression across distinct tissue types (Figure 2). For instance, RE1‐silencing transcription factor (REST) plays a critical regulatory role in cardiac pathology. Genetic deletion of REST exacerbates myocardial fibrosis and inflammation following pressure overload and this phenotype driven in part by the secondary upregulation of UCHL1, which actively promotes pathological hypertrophy [27]. Significantly, pharmacological inhibition of UCHL1 via LDN‐57444 in REST conditional knockout mice successfully mitigates hypertrophic fibrosis and inflammatory signaling by dampening the NF‐κB and STAT3 pathways [27].

In parallel, the NF‐κB pathway itself serves as a prominent upstream regulator of *UCHL1* transcription, though its effects appear to be highly context‐dependent. In models of renal podocyte injury, immune complex‐mediated inflammation activates NF‐κB, which directly drives *UCHL1* gene expression [28]. Similarly, investigations into cardiac remodeling identify NF‐κB as a direct transcriptional activator of *UCHL1* [29]. Conversely, early foundational studies indicated that under certain experimental conditions, NF‐κB activation can suppress *UCH‐L1* promoter transcription, underscoring a complex, context‐specific regulatory mechanism [30].

Beyond these pathways, specificity protein 1 (Sp1) has also been implicated in UCHL1 modulation. In an H_2_O_2_‐induced model of age‐related hearing loss, the Sp1‐mediated suppression of UCHL1 transcription markedly attenuates cellular senescence and oxidative stress within cochlear hair cells [31]. Finally, epigenetic mechanisms significantly influence UCHL1 expression in malignant contexts. In triple‐negative breast cancer (TNBC), ten‐eleven translocation 1 (TET1) and TET3 actively enhance *UCHL1* transcription by demethylating its promoter; this locus activation subsequently sustains Krüppel‐like factor 5 (KLF5) protein levels via UCHL1‐mediated deubiquitination to drive cancer progression [14].

### 3.2 Epigenetic Regulation

DNA methylation represents a primary epigenetic mechanism governing UCHL1 expression, particularly within malignant contexts. Aberrant epigenetic silencing of UCHL1 via promoter CpG island hypermethylation serves as a strong prognostic indicator, predicting advanced disease development and poor clinical outcomes in renal cell carcinoma [32]. Similarly, in prostate cancer (PCa), *UCHL1* silencing has been definitively linked to promoter hypermethylation, which correlates with a profound reduction in UCHL1 transcript and protein levels in malignant tissues compared to adjacent, benign prostate epithelium [33]. Furthermore, the loss of UCHL1 expression driven by CpG promoter hypermethylation is significantly associated with metastatic progression in gastroenteropancreatic neuroendocrine tumors (GEP‐NETs) [34]. This epigenetic silencing pattern extends to nasopharyngeal carcinoma as well, where promoter hypermethylation has been identified as the predominant molecular mechanism responsible for downregulating UCHL1 expression and disrupting its downstream tumor‐suppressive pathways [35].

### 3.3 Noncoding RNA‐Mediated Regulation

Posttranscriptional networks driven by noncoding RNAs add an additional layer of precision to UCHL1 expression control. For instance, miR‐922 directly inhibits the translation of UCHL1 mRNA by binding to its 3′ prime untranslated region, thereby significantly reducing endogenous UCHL1 protein expression levels. This microRNA‐mediated depletion of UCHL1 subsequently disrupts the delicate homeostatic balance of tau protein phosphorylation, ultimately accelerating the tau pathology characteristic of Alzheimer's disease [36]. Conversely, long noncoding RNAs (LncRNAs) can actively enhance UCHL1 expression. In dopaminergic neurons, long noncoding antisense RNA (AS‐*Uchl1*) acts as a translational upregulator by pairing with *Uchl1* mRNA to promote its translation, thereby increasing UCHL1 protein abundance and supporting neuronal homeostasis [37].

### 3.4 Posttranslational Modifications

Beyond transcriptional and epigenetic control, UCHL1 function is dynamically modulated at the protein level by an array of posttranslational modifications that alter its enzymatic activity, stability, and cellular localization. For example, S‐nitrosylation of UCHL1 directly disrupts its core hydrolase activity. This modification impairs UPS function, linking nitric oxide‐mediated oxidative stress to the dysregulation of neuronal proteostasis [38]. Similarly, high‐throughput proteomic analyses of Alzheimer's disease brain tissue have identified UCHL1 as a primary target of pathological oxidation. Extensive oxidative modification of its critical cysteine residues severely compromises both its protein solubility and its catalytic activity [39, 40].

UCHL1 abundance is also tightly regulated by the ubiquitination machinery itself. The E3 ubiquitin ligase Parkin‐mediated K63‐linked chains can modify UCHL1, targeting the enzyme for degradation via the autophagy‐lysosome pathway rather than the classic proteasome, and this cross‐system targeting acts as a major checkpoint to downregulate steady‐state UCHL1 levels and functional activity [41]. Furthermore, UCHL1 partitions into distinct biophysical compartments existing as both a soluble cytosolic protein and a distinct membrane‐bound species termed UCHL1^M^ [26]. This membrane association is driven by lipid modification, specifically C‐terminal farnesylation, which anchors the protein to intracellular lipid bilayers, such as the endoplasmic reticulum. Pathologically, elevated UCHL1^M^ expression in transfected models correlates closely with the stabilization and accumulation of α‐synuclein, driving subsequent neurotoxicity and Parkinson's disease progression [26].

Taken together, these upstream regulatory mechanisms demonstrate that UCHL1 is subject to an intricate network of transcriptional, epigenetic, and posttranslational controls. Collectively, these inputs dictate its highly tissue‐specific expression patterns, baseline stability, and ultimate functional outcomes in both homeostatic and diseased states.

## 4 UCHL in Human Disease

### 4.1 Neurological Disorders

**FIGURE 3:**
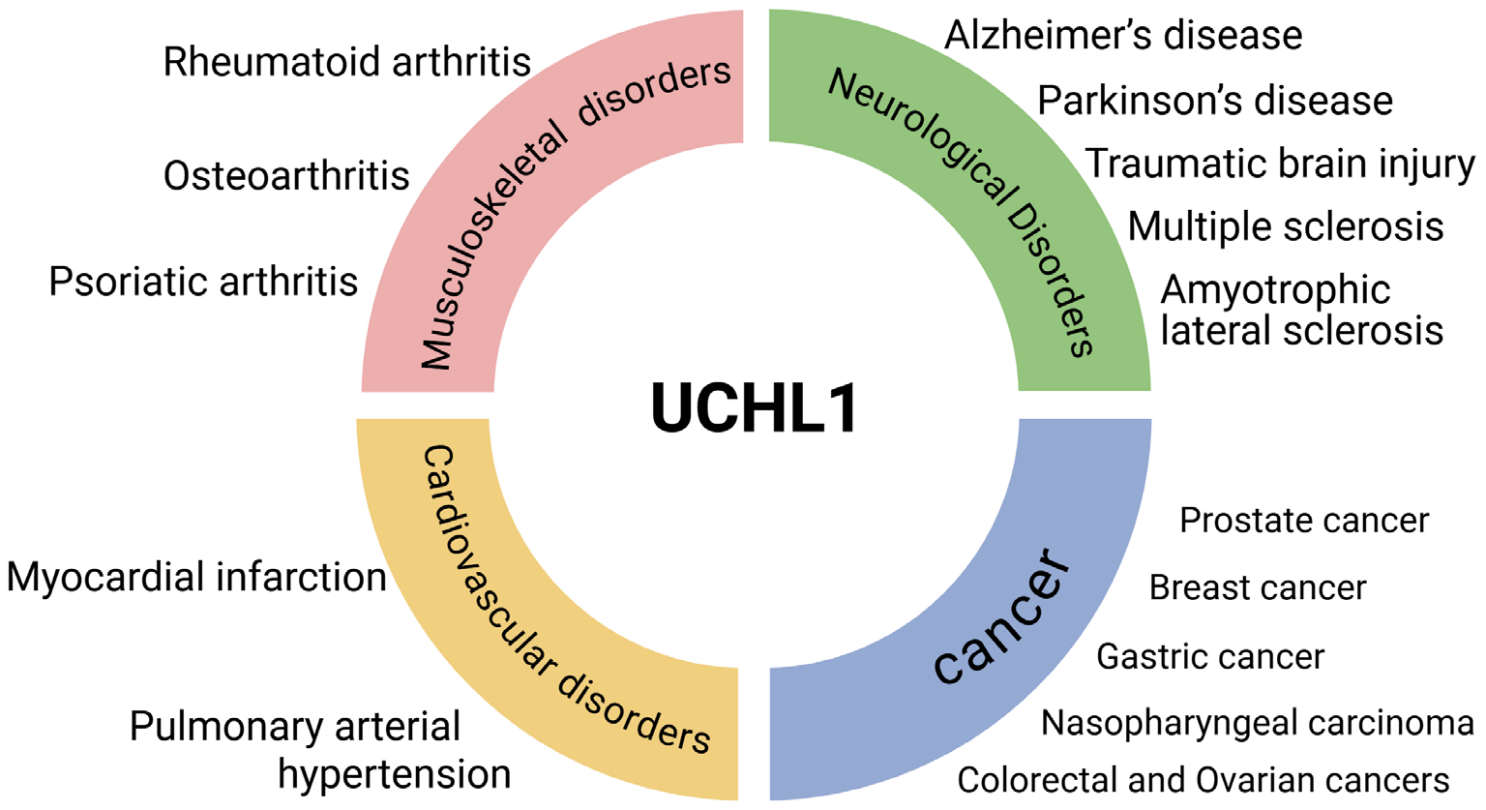
UCHL1‐associated pathology. The pathological role of UCHL1 in multisystem diseases, including cancer, neurological, musculoskeletal and cardiovascular disorders, is summarized.

The relevance of UCHL1 to neurological disorders must be interpreted within the broader structural and functional framework of ubiquitin‐dependent proteostasis. Because neurons are long‐lived, postmitotic cells with virtually no regenerative capacity, they are exquisitely dependent on flawless protein quality‐control networks. In neurodegenerative diseases, the accumulation of misfolded or aggregation‐prone proteins can rapidly overwhelm these cellular degradation pathways, culminating in the formation of cytotoxic, ubiquitin‐positive inclusions, the impairment of baseline proteasomal activity, and the disruption of critical crosstalk between the UPS and the autophagy–lysosome system. Consequently, UCHL1 is mechanistically relevant to neurodegeneration not merely because its expression levels fluctuate in diseased tissues, but because it actively governs the core machinery of ubiquitin recycling, protein turnover, and stress‐related signaling pathways that dictate neuronal homeostasis and survival [11, 42, 43] (Figure 3).

#### 4.1.1 Alzheimer's Disease (AD)

The hallmark pathological features of Alzheimer's disease (AD) comprise extracellular amyloid‐β (Aβ) plaques and intracellular neurofibrillary tangles (NFTs). The neurotoxic Aβ peptide is derived from the amyloid precursor protein (APP) via sequential proteolytic cleavage by beta‐site amyloid precursor protein‐cleaving enzyme 1 (BACE1) and the γ‐secretase complex. Pharmacological inhibition or genetic deletion of UCHL1 markedly elevates BACE1 protein expression and subsequent Aβ accumulation; in contrast, transgenic overexpression of UCHL1 significantly reduces Aβ burden and rescues spatial memory deficits in APP/PS1 mice [44, 45]. Parallel to its control over amyloidogenesis, UCHL1 actively protects against tau‐mediated pathology rather than accelerating NFT formation. Knockdown of *UCHL1* increases the accumulation of hyperphosphorylated tau and exacerbates the overall NFT burden, whereas its upregulation reduces tau hyperphosphorylation [46]. Mechanistically, the absence of UCHL1 impairs the HDAC6‐mediated aggresome processing of tau protein, a pathway proposed to act as a protective autophagic sequestration response, thereby leaving soluble, hyperphosphorylated tau intermediates free to exert heightened neurotoxicity [47]. In addition to driving these proteinopathies through downstream targets, UCHL1 itself serves as a direct casualty of the AD microenvironment. It undergoes extensive posttranslational modifications, including pathological oxidation and S‐nitrosylation, within the brains of AD patients; these structural modifications drastically reduce UCHL1 solubility and disrupt its deubiquitinating activity within the UPS, creating a feed‐forward loop that accelerates macro‐aggregate accumulation and subsequent neuronal death [39, 40]. Finally, UCHL1 exerts a critical immunomodulatory role against AD‐associated neuroinflammation. Direct exposure to Aβ significantly downregulates both the neuroprotective receptor TREM2 and endogenous UCHL1 in glial models. Notably, administration of recombinant UCHL1 restores TREM2 expression levels and normalizes the aberrant secretion of pro‐inflammatory cytokines, including IL‐6 and TNF, highlighting a potent, multi‐pronged neuroprotective and anti‐inflammatory modality for UCHL1 in the degenerating brain [48].

#### 4.1.2 Parkinson's Disease (PD)

Parkinson's disease (PD) is pathologically characterized by the progressive loss of dopaminergic neurons in the substantia nigra and the intracellular accumulation of α‐synuclein into insoluble aggregates known as Lewy bodies. Because α‐synuclein is an inherently aggregation‐prone protein whose clearance relies heavily on cellular quality‐control pathways, systemic dysfunction of the UPS and the autophagy–lysosome pathway lies at the core of PD pathobiology. Within this framework, UCHL1 operates as a key modulator of pathogenic pathways shared by both familial and sporadic forms of the disease [49]. Historically, a rare missense mutation in the *UCHL1* gene resulting in an isoleucine‐to‐methionine substitution at position 93 (I93M) was identified in a family with autosomal dominant, early‐onset familial PD, designating the *PARK5* locus [50]. Although subsequent genetic screenings have confirmed that *UCHL1* mutations represent an uncommon cause of hereditary PD globally, the discovery of the I93M variant was highly foundational, establishing a definitive mechanistic link between disrupted ubiquitin biology, proteostatic failure, and α‐synuclein‐driven neurodegeneration.

A principal molecular mechanism connecting UCHL1 to PD pathogenesis is its regulation of chaperone‐mediated autophagy (CMA), a highly selective lysosomal degradation pathway. Under physiological conditions, targeted substrate proteins displaying a pentapeptide KFERQ‐like motif are recognized by the cytosolic chaperone heat shock cognate 71 kDa protein (Hsc70) and co‐chaperones (such as Hsp90), which deliver them to the lysosomal membrane for translocation via the receptor lysosome‐associated membrane protein 2A (LAMP‐2A) [51]. CMA is heavily implicated in PD because wild‐type α‐synuclein is a primary substrate for this clearance pathway. Mechanistic dissections have revealed that UCHL1 physically interacts with the core components of the CMA machinery, including LAMP‐2A, Hsc70, and Hsp90, thereby functioning as a homeostatic rheostat for α‐synuclein degradation [52, 53]. Familial PD‐associated mutant or oxidatively modified UCHL1 can aberrantly interact with the CMA machinery, impair lysosomal degradation, and increase α‐synuclein accumulation [52]. Therefore, UCHL1 is relevant to PD not simply as a genetic association, but as a regulator of proteostasis, CMA function, and α‐synuclein turnover.

#### 4.1.3 Traumatic Brain Injury (TBI)

Because UCHL1 is highly enriched within the neuronal cytoplasm, its secondary release into the peripheral blood or cerebrospinal fluid (CSF) serves as a direct proxy for acute neuronal cell injury, compromised axonal membrane integrity, or necrotic cell death. Clinically, UCHL1, in combination with glial fibrillary acidic protein (GFAP), has received FDA clearance as a blood‐based diagnostic biomarker panel for the acute evaluation of suspected mild TBI [54, 55]. The clinical integration of this serological assay primarily serves to aid clinicians in determining the necessity of head computed tomography (CT) scans, thereby reducing unnecessary radiation exposure. Extensive observational studies demonstrate that elevated circulating concentrations of UCHL1 correlate strongly with overall injury severity, the presence of CT‐detectable acute intracranial lesions, and long‐term functional clinical outcomes, validating its translational utility as a highly sensitive marker of real‐time neuronal damage [54, 56].

#### 4.1.4 Multiple Sclerosis (MS)

Multiple sclerosis (MS) is a chronic, immune‐mediated demyelinating disease of the central nervous system characterized by neuroinflammation, focal demyelination, and progressive axonal degeneration. Recent clinical investigations evaluating serum and CSF biomolecules in MS cohorts have demonstrated that neurofilament light chain (NfL) and GFAP exhibit highly consistent associations with real‐time neuroaxonal injury and reactive astrogliosis, respectively [57]; however, the standalone diagnostic and prognostic utility of UCHL1 remains less established. Thus, within the heterogeneous landscape of MS pathology, UCHL1 is more appropriately conceptualized as a downstream surrogate marker of secondary neuronal or axonal damage occurring in the wake of chronic neuroinflammation, rather than as an active, primary driver of the demyelinating process itself. Beyond its passive release from degenerating axons, UCHL1‐positive microglial extracellular vesicles detected in peripheral blood have recently been proposed as novel, adjunctive immune‐neural biomarkers capable of reflecting central neuroinflammatory states [58]; however, the precise pathological significance and diagnostic sensitivity of these vesicular structures require extensive further validation in longitudinal clinical trials.

#### 4.1.5 Amyotrophic Lateral Sclerosis (ALS)

Amyotrophic lateral sclerosis (ALS) is a devastating, progressive neurodegenerative disease characterized by the selective loss of upper and lower motor neurons, a process driven by severely impaired proteostasis, the accumulation of ubiquitinated protein inclusions, and systemic dysfunction of cellular clearance pathways. The UPS failure is particularly central to ALS pathobiology, as abnormal protein macro‐aggregates and ubiquitin‐positive intracellular inclusions represent defining pathological features of degenerating motor neurons. Multiple key proteins implicated in ALS pathogenesis (e.g., TDP‐43, SOD1, and FUS) are prone to pathological misfolding, aggregate formation, and aberrant degradation kinetics via the crosstalk between the UPS and the autophagy–lysosome pathways. Within this context of proteostatic collapse, UCHL1 has emerged as a promising candidate biomarker in both the serum and CSF of ALS patients with elevated circulating levels of the enzyme are thought to accurately reflect real‐time mechanical neuronal injury or the degree of disease‐associated proteostatic stress [59]. While its utility as a liquid biopsy surrogate marker continues to gather clinical interest, direct mechanistic evidence demonstrating that UCHL1 actively or causally contributes to the underlying molecular pathogenesis of ALS remains limited.

### 4.2 Cancer

The divergent roles of UCHL1 across various malignancies can be reconciled through two primary, opposing molecular mechanisms: widespread epigenetic silencing, which abrogates its intrinsic tumor‐suppressive properties in specific tissue types, and targeted substrate stabilization, which hyperactivates oncogenic signaling cascades in others. Consequently, UCHL1 cannot be simplistically classified as a universal oncogene or a classic tumor suppressor. Instead, it functions as a context‐dependent proteostatic regulator whose final phenotypic output is dictated by promoter methylation status, cellular lineage, and the specific repertoire of downstream substrates available within the tumor microenvironment.

Accumulating studies demonstrate that distinct members of the UCH enzyme family modulate diverse signaling pathways critical to malignant transformation and metastatic dissemination [60]. This operational divergence is clearly illustrated by the opposing roles UCHL1 plays in breast versus prostate malignancies. In breast cancer, UCHL1 is frequently overexpressed and functions as a key oncogenic driver. Mechanistic investigations have revealed that elevated UCHL1 directly stabilizes estrogen receptor α (ERα) and hyperactivates the PI3K/AKT signaling axis, thereby accelerating tumor cell proliferation, driving local tissue invasion, and conferring therapeutic resistance to standard endocrine therapies [61]. Conversely, in prostate cancer (PCa), the *UCHL1* locus undergoes profound transcriptional silencing driven by extensive promoter hypermethylation. Experimental re‐expression of UCHL1 in PCa models successfully suppresses cell proliferation and restricts in vivo tumor growth. This tumor‐suppressive phenotype is mechanistically executed through the robust activation of the p53/p14(ARF) signaling axis alongside the concomitant downregulation of oncogenic PI3K/AKT signaling [33]. Together, these findings highlight how the microenvironmental context and lineage background completely invert UCHL1's functional consequences.

Similarly, colorectal and ovarian carcinomas frequently exhibit profound epigenetic silencing of UCHL1, and this loss of expression removes key homeostatic checkpoints, thereby supporting accelerated tumor expansion and enhanced cell survival [62]. In contrast, UCHL1 shifts to an active promoter of malignancy in other anatomical contexts. For example, in gastric cancer cell lines, the functional ablation or decreased expression of UCHL1 significantly impedes cell proliferation, migration, and invasive capacity [63]. Furthermore, in high‐grade neuroendocrine lung cancers, UCHL1 is strongly upregulated and tightly correlated with highly aggressive clinical behaviors. In these neuroendocrine models, *UCHL1* knockdown robustly restricts cell proliferation and compromises metastatic dissemination, highlighting its potential utility as both an adverse prognostic indicator and a viable therapeutic target [14, 35]. Ultimately, the dual role of UCHL1 across the oncogenic spectrum reflects a complex equilibrium between cell‐heritable epigenetic regulation and lineage‐specific substrate signaling, emphasizing that any translational application requires a highly tumor‐context‐specific interpretation.

### 4.3 Cardiovascular Disorders

Beyond its role in cell proliferation, UCHL1 serves as a critical modulator of fibroblast activation and vascular signaling. In both cardiac and vascular injury models, UCHL1 fosters transforming growth factor‐β (TGF‐β)/NF‐κB‐linked fibrotic pathways. Consequently, its targeted inhibition or genetic ablation significantly restricts pathological fibroblast activation and mitigates adverse postmyocardial infarction remodeling [29, 64]. Furthermore, UCHL1 contributes to vascular remodeling under chronic pressure states. In models of pulmonary arterial hypertension (PAH), the pharmacological inhibition or ablation of *UCHL1* markedly ameliorates clinical phenotypes. Mechanistically, this therapeutic effect is driven by accelerating the K48‐linked polyubiquitination and subsequent proteasomal degradation of AKT1 within endothelial cells, thereby suppressing aberrant endothelial proliferation and vascular thickening [23]. Because adverse tissue remodeling, fibroblast hyperactivation, persistent inflammatory signaling, and altered matrix repair responses represent shared pathogenic hallmarks of several chronic musculoskeletal disorders, these cardiovascular insights carry broader translational implications.

### 4.4 Musculoskeletal Disorders

#### 4.4.1 Rheumatoid Arthritis (RA)

In rheumatoid arthritis (RA), emerging evidence points to a multi‐faceted role for UCHL1 at the intersection of synovial innervation, persistent inflammation, and articular bone remodeling. Early histological investigations reported a marked depletion of UCHL1/PGP9.5‐positive intra‐articular nerve fibers within the inflamed RA synovium, implicating altered sensory innervation and dysregulated neurogenic inflammatory pathways in chronic joint pathology [65].

At the cellular and molecular level, recent mechanistic insights highlight UCHL1 as a potent modulator of both myeloid‐driven inflammation and periarticular bone destruction. Because UCHL1 directly governs the NACHT domain of the NLRP3 inflammasome to regulate macromolecular assembly and subsequent IL‐1β production in human macrophages, this noncanonical scaffolding pathway likely exacerbates synovial inflammation, though its direct validation within primary RA synovial tissue cohorts remains an area of active investigation [12]. Furthermore, UCHL1 contributes to the amplification of joint inflammation by actively promoting macrophage polarization toward the pro‐inflammatory M1 phenotype [66]. Conversely, within the bone microenvironment, UCHL1 functions as a critical homeostatic checkpoint that restricts excessive bone resorption. During RANKL‐mediated osteoclastogenesis, UCHL1 deubiquitinates and stabilizes the transcriptional coactivator with PDZ‐binding motif (TAZ), which downregulates NFATc1 to impair osteoclast differentiation [21]. This protective axis is further supported by a negative feedback loop wherein UCHL1 stabilizes CD13 and enhances the shedding of soluble CD13, which subsequently suppresses downstream MAPK signaling to halt osteoclast differentiation [20]. Taken together, these contrasting mechanisms place UCHL1 at a pivotal regulatory juncture controlling the persistent synovitis, macrophage activation, and aggressive bone erosion that define RA pathogenesis.

#### 4.4.2 Osteoarthritis (OA)

The pathological hallmarks of osteoarthritis (OA) are primarily characterized by progressive articular cartilage destruction, subchondral bone sclerosis, and low‐grade chronic synovitis. Within the joint, chondrocytes permanently inhabit a highly hypoxic, mechanically loaded avascular niche where HIF‐1α acts as a master regulator to sustain baseline glycolysis and cell survival. UCHL1 directly intersects with this survival mechanism by limiting the ubiquitination and subsequent proteasomal degradation of HIF‐1α, thereby significantly reducing chondrocyte apoptosis in vitro and supporting robust extracellular matrix homeostasis within chondrogenic differentiation systems. These data functionally connect a traditionally neuron‐centric deubiquitinating enzyme to canonical cartilage stress pathways, highlighting its potential translational relevance in mitigating OA cartilage degeneration [67]. Furthermore, targeted CRISPR‐mediated activation of *UCHL1* enhances the chondrogenesis of mesenchymal stem cells while simultaneously downregulating key matrix degradation markers. This indicates that UCHL1 actively supports cartilage anabolism and matrix synthesis within developmental and regenerative contexts [19]. This protective, anabolic role in the joint space stands in sharp contrast to the enzyme's well‐documented catabolic, pro‐fibrotic actions observed in certain cardiovascular and systemic fibrotic settings. Ultimately, these divergent phenotypes reinforce the absolute necessity for highly context‐specific, tissue‐targeted therapeutic modulation when targeting UCHL1 pathways in chronic joint diseases.

Beyond its direct protective effects on articular chondrocytes, UCHL1 serves as a critical rheostat regulating the biological characteristics of osteoclasts and the structural integrity of the subchondral bone. High‐throughput proteomic profiling has revealed that UCHL1 expression is markedly upregulated during RANKL‐induced osteoclastogenesis, establishing it as a necessary component for baseline myeloid differentiation [68]. However, rather than acting as a simple driver of bone resorption, UCHL1 predominantly functions to fine‐tune this process, serving as an intrinsic brake against excessive osteoclast activation through two major molecular axes: (1) The sCD13 negative feedback loop, in which RANKL stimulation induces the secretion of soluble CD13 (sCD13) via the downstream ERK/p38 MAPK signaling cascade. In turn, sCD13 acts extracellularly to inhibit osteoclast formation and attenuate bone erosion. UCHL1 directly participates in this protective negative feedback loop by stabilizing the CD13 machinery, offering a discrete extracellular entry point to restrain osteoclast hyperactivation during inflammatory bone loss [20]. (2) The TAZ/NFATc1 axis, in which UCHL1 negatively controls the trajectory of osteoclastogenesis by binding to and stabilizing the transcriptional coactivator with PDZ‐binding motif (TAZ; also known as WWTR1). By preventing its degradation, UCHL1 allows TAZ to repress NFATc1, the master transcriptional regulator of osteoclast differentiation, as well as its downstream osteoclast effector genes, thereby controlling pathological bone resorption [21]. The physiological consequence of disrupting this regulatory equilibrium is underscored by recent genetic loss‐of‐function studies. The absolute absence or deletion of UCHL1 significantly accelerates osteoclast differentiation and exacerbates bone resorption, a phenotype mechanistically driven by the secondary hyperactivation of the pro‐survival AKT signaling pathway [69]. Together, these findings demonstrate that while UCHL1 expression increases during initial myeloid commitment, it paradoxically functions as a critical homeostatic tuner that prevents catastrophic, over‐activated bone destruction within the subchondral microenvironment.

#### 4.4.3 Psoriatic Arthritis (PsA)

At present, direct empirical data linking UCHL1 to the pathogenesis of psoriatic arthritis (PsA) remain scarce. Classic PsA pathology is primarily driven by the systemic IL‐23/IL‐17 axis and downstream signal transducer and activator of transcription 3 (STAT3) signaling cascades [70]; however, direct mechanistic evidence placing UCHL1 within this specific signaling network has yet to be fully established. Nevertheless, given its well‐documented role in orchestrating NF‐κB activation and NLRP3 inflammasome assembly within myeloid lineages, alongside its critical function in chondrocyte stress adaptation within the hypoxic joint environment, UCHL1 stands as a candidate molecule at the intersection of mechanical stress, chronic inflammation, and entheseal or synovial tissue remodeling.

## 5 Conclusion and Future Perspectives

UCHL1 has evolved conceptually from a line‐restricted, neuron‐enriched deubiquitinating enzyme into a pleiotropic regulator implicated in a vast array of pathological processes across multiple organ systems. As discussed in this review, UCHL1 orchestrates protein quality control, inflammatory signaling cascades, metabolic stress adaptations, and cellular fate decisions in a highly context‐dependent manner. Within the central nervous system, it dictates APP/Aβ processing, tau hyperphosphorylation kinetics, α‐synuclein clearance, and neuroinflammatory responses, a functional prominence underscored by its clinical translation as an FDA‐approved serum biomarker for traumatic brain injury. In oncology, UCHL1 displays a complex duality, exerting either oncogenic or tumor‐suppressive phenotypes that are strictly contingent upon the localized epigenetic landscape and lineage‐specific substrate availability. Through these mechanisms, it modulates critical axes including ERα, PI3K/AKT, p53/p14(ARF), HIF‐1α, and various chromatin‐remodeling complexes. Furthermore, recent paradigm‐shifting evidence expands its reach into cardiovascular fibrosis, pulmonary vascular remodeling, articular chondrocyte homeostasis, subchondral osteoclastogenesis, macrophage M1/M2 polarization, and NLRP3 inflammasome assembly, highlighting its profound systemic relevance to chronic musculoskeletal and inflammatory diseases.

Despite these substantial advancements, several fundamental questions remain unresolved. First, while UCHL1 exhibits distinct tissue‐ and stimulus‐specific functionalities, the precise molecular switches governing these divergent outcomes, particularly its contrasting roles in malignant transformation versus fibrotic tissue regeneration, remain to be fully elucidated. Second, the comprehensive landscape of UCHL1 substrates and physical interacting partners is far from complete. Emerging high‐throughput proteomics and structural biology datasets hint at noncanonical modalities, including novel scaffolding properties and potential ligase activities, that require rigorous mechanistic validation. Third, modulating UCHL1 therapeutically remains a major hurdle. Although first‐generation small‐molecule inhibitors like LDN‐57444 are ubiquitous in preclinical discovery, they lack the target selectivity and pharmacokinetic profile required for clinical translation. There is an urgent, unmet need for highly specific UCHL1 small‐molecule inhibitors or degraders. Finally, the discovery of UCHL1's regulatory role in musculoskeletal conditions, and at the immune‐metabolic interface necessitates a thorough evaluation of this enzyme as a novel therapeutic target or diagnostic biomarker for chronic, destructive joint pathologies.

Looking forward, integrating single‐cell transcriptomics, spatial proteomics, high‐dimensional ubiquitin chain mapping, and sophisticated in vivo conditional genetic models will be paramount to dissecting the precise spatiotemporal activity patterns of UCHL1. Deciphering how this enzyme senses and responds to localized hypoxia, persistent inflammation, epigenetic modifications, and metabolic stress will provide the conceptual framework necessary for rational drug discovery. Ultimately, as an intricate molecular hub deeply embedded in pathologies ranging from neurodegeneration and cancer to vascular remodeling and bone erosions, capturing the regulatory complexity of UCHL1 will be the key to translating these basic science insights into meaningful clinical interventions.

## Author Contributions

**Xiu Zhong:** writing – review and editing. **Adrian A. Achuthan:** writing – review and editing. **Jianjun Li:** writing – review and editing. **Ru Feng:** writing – original draft.

## Conflicts of Interest

The authors declare no conflicts of interest.

## Data Availability

Data sharing not applicable to this article as no datasets were generated or analysed during the current study.
